# Dosimetric comparison of Helical Tomotherapy and Gamma Knife Stereotactic Radiosurgery for single brain metastasis

**DOI:** 10.1186/1748-717X-1-26

**Published:** 2006-08-03

**Authors:** José A Peñagarícano, Yulong Yan, Chengyu Shi, Mark E Linskey, Vaneerat Ratanatharathorn

**Affiliations:** 1 Associate Professor of Radiation Oncology, University of Arkansas for Medical Sciences, Little Rock, Arkansas 72205, USA; 2 Adjunct Assistant Professor of Radiation Oncology, Cancer Therapy and Research Center, San Antonio TX 78229, USA; 3 Associate Professor and Chair, Department of Neurological Surgery, University of California, Irvine Medical Center, Orange, CA 92868, USA; 4 Professor and Chair of Radiation Oncology, University of Arkansas for Medical Sciences, Little Rock, Arkansas 72205, USA

## Abstract

**Background:**

Helical Tomotherapy (HT) integrates linear accelerator and computerized tomography (CT) technology to deliver IMRT. Targets are localized (i.e. outlined as gross tumor volume [GTV] and planning target volume [PTV]) on the planning kVCT study while daily MVCT is used for correction of patient's set-up and assessment of inter-fraction anatomy changes. Based on dosimetric comparisons, this study aims to find dosimetric equivalency between single fraction HT and Gamma Knife^® ^stereotactic radiosurgery (GKSRS) for the treatment of single brain metastasis.

**Methods:**

The targeting MRI data set from the GKSRS were used for tomotherapy planning. Five patients with single brain metastasis treated with GKSRS were re-planned in the HT planning station using the same prescribed doses. There was no expansion of the GTV to create the PTV. Sub-volumes were created within the PTV and prescribed to the maximum dose seen in the GKSRS plans to imitate the hot spot normally seen in GKSRS. The PTV objective was set as a region at risk in HT planning using the same prescribed dose to the PTV periphery as seen in the corresponding GKSRS plan. The tumor volumes ranged from 437–1840 mm^3^.

**Results:**

Conformality indices are inconsistent between HT and GKSRS. HT generally shows larger lower isodose line volumes, has longer treatment time than GKSRS and can treat a much larger lesion than GKSRS. Both HT and GKSRS single fraction dose-volume toxicity may be prohibitive in treating single or multiple lesions depending on the number and the sizes of the lesions.

**Conclusion:**

Based on the trend for larger lower dose volumes and more constricted higher dose volumes in HT as compared to GKSRS, dosimetric equivalency was not reached between HT and GKSRS.

## Background

For patients with single brain metastasis, the addition of surgical resection or radiosurgery to whole brain radiation therapy improves survival [1,2]. In Gamma Knife^® ^stereotactic radiosurgery (GKSRS), a single fraction of radiation is used to treat metastatic lesions in the brain. There appears to be a fine line between treatment success and the predominant form of late-toxicity from GKSRS, radiation necrosis [3]. Helical tomotherapy is an emerging technology based mainly on the linkage and integration of known and widely-used technology in radiation oncology into a single system, i.e. a linear accelerator and computed tomography, allowing precise daily targeting of IMRT using megavoltage CT (MVCT) guidance.

In this study we will compare dosimetric plans between GKSRS and single fraction helical tomotherapy (HT) for five patients with single brain metastasis by examining the PTV coverage by the prescribed isodose surface, and the high- and low-dose spillage volumes. As Gamma Knife^® ^is an accepted technology for stereotactic radiosurgery, our goal is not show a superiority of one technology over the other but to see if dosimetric equivalency between the two technologies can be achieved.

## Methods

### Patients

Five patients with single brain metastasis were selected at random from the pool of previously treated patients with GKSRS. These were planned for single fraction radiosurgery using the Tomotherapy Hi-ART system.

### Stereotactic Radiosurgery

The Gamma Knife^® ^Model B by Elekta (Norcross, Georgia) was used in this study. The Gamma Knife^® ^device and the involved radiosurgery technique have been described previously [4,5]. Briefly, patients offered GKSRS have a Karnofsky Performance Status (KPS) equal or larger than 70 points and a single brain lesion < 3.5 cm treatment volume or volume of the prescribed isodose surface of a maximum of 30 cc. Patients with extracranial disease were accepted if it was felt that their life expectancy would be at least 6 months. On the day of the GKSRS procedure, a stereotactic frame was placed under local anesthesia and a three dimensional contrast enhanced MRI of the entire brain was obtained. The MRI was reviewed by neuro-radiology to confirm the presence of a single brain metastasis. Then a contrast enhanced 3D SPGR (Spoiled Gradient Recalled sequence) MRI was obtained through the area of interest (targeting MRI) with axial images every 1 mm. The GTV was outlined in the targeting MRI. No expansion of the GTV was allowed to create the PTV. One or more isocenters were planned to create isodose lines conforming to the three-dimensional PTV. Tumor volumes ranged from 437 to 1840 mm^3^. Radiosurgery doses ranged from 16 to 20 Gy normalized to the 50% isodose line in all five patients.

### Hi-ART Tomotherapy system

In the Hi-ART Tomotherapy (Madison, Wisconsin) system, a 6-MV linear accelerator is mounted on a ring gantry in a CT configuration [6-8]. Opposite the linear accelerator is an array of Xenon detectors capable of measuring exit dose. The beam in a helical tomotherapy system is collimated by a pneumatically driven multi-leaf collimator that produces a fan beam with width of 0.53 to 5 cm. Patients lay on the table that moves through the ring gantry while the gantry is rotating. That results in a helical form of radiation delivery, minimizing junctional problems. The helical tomotherapy system is capable of treatment delivery and acquisition of mega voltage CT (MVCT) images with clinically satisfactory image quality and resolution. By taking a CT scan before treatment, physicians are able to verify the patient's anatomy, including tumor characteristics and critical structures. This allows them to quickly update any changes in the patient's position [9-11]. The port set-up is indexed to any fixed internal structures, such as bony landmarks, rather than to external skin markings or thermoplastic mask fiducials as is currently done with linear accelerator-based IMRT delivery. HT has no externally moving parts, except for the treatment table, so there is no chance for collision.

### HT planning

The targeting MRI data set and regions of interest files were transferred to the Tomotherapy planning station via DICOM-RT protocol. The details of the inverse planning algorithm used in the Tomotherapy unit have been described before [12]. The optimization is guided using several parameters, which have been described in the literature [13]. The user defines the prescription, the jaw opening, the modulation factor (MF), the pitch, and the resolution of the calculation grid. Jaw opening, pitch and MF were 0.53 centimeter, 0.200 and 2.0 for all patients, respectively. The choice of jaw width, pitch and modulation factor were chosen on the basis of obtaining a set of optimization parameters that would allow sufficient field overlap per gantry rotation. This in turn will allow sufficient modulation of the beam within the target.

No expansion of the GTV was allowed to create the PTV. Dose and dose-volume objectives can be defined for the PTV and the organs at risk with differential penalties. In order to create inhomogeneity within the PTV, sub-volumes were created within the PTV. These sub-volumes were then prescribed the maximum dose as seen in the corresponding GKSRS plan. The PTV objective was defined as a organ at risk in order to attempt to maintain the periphery dose as seen in the corresponding GKSRS plan.

### Dosimetric analysis

In each patient, dose volumes were calculated at dose levels ranging from 5–40 Gy at 5 Gy volume increments. In addition, the coverage and conformality index as described by Paddick [14] and the total treatment time (beam-on time) were obtained from the corresponding planning stations for each plan.

## Results

**Figure 1:**
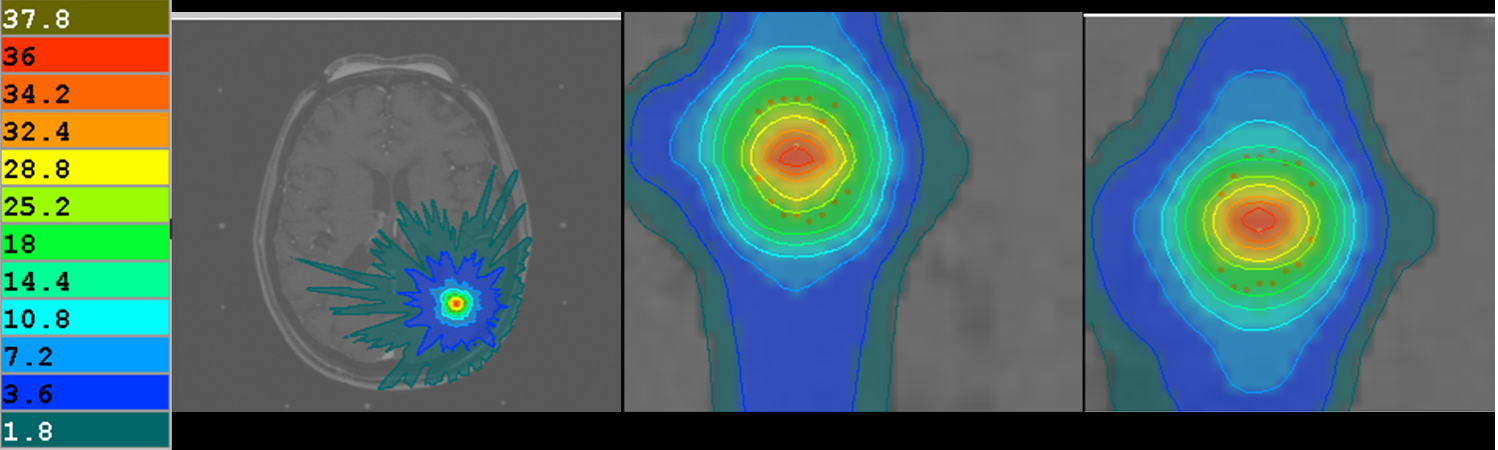
Represents the Tomotherapy dose distribution (in Gy) for one of the five presented patients.

**Figure 2:**
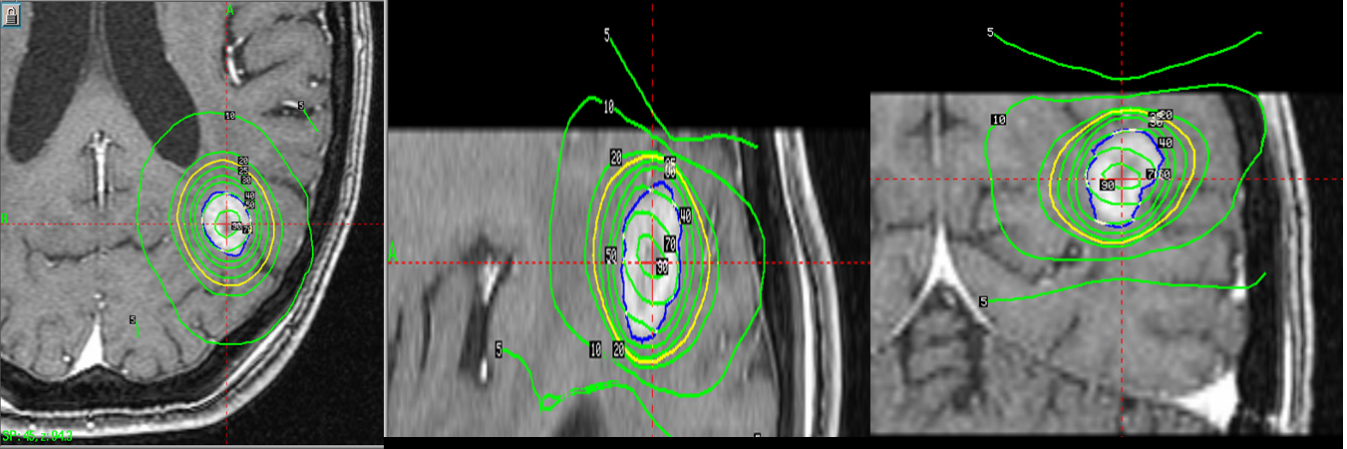
Represents the Gamma Knife dose distribution (in percent of the prescribed dose) for one of the five presented patients.

Figure 1 and figure 2 show HT and GKSRS dose distribution for one of the presented patients. Results are summarized in Tables 1, 2, 3, 4, 5. In these tables patient order reflects increasing tumor volume. For patient #1, the higher dose volumes (15–40 Gy) were smaller for the HT plans but the lower dose volumes (5–10 Gy) were larger in HT plans by 1.5 and 1.06 times, respectively. The conformality indices (CI) and the beam-on treatment time (T) are 0.577 & 0.597 and 43.00 & 50.77 minutes for the HT and GKSRS plans, respectively. For patient #2, the high dose volumes from 25–35 Gy was larger for the GKSRS plans and the reverse was true for the low dose volume from 5–15 Gy (range: 1.49 to 2.69 times larger). CI and T for the HT plans as compared to GKSRS were 0.562 & 0.618 and 34.00 & 21.00 minutes, respectively. For patient #3 with a brain stem lesion, all existing dose volumes from 5–30 Gy were larger in the HT plans (1.37 to 2.89 times larger). CI and T for the HT plans as compared to GKSRS were 0.603 & 0.593 and 30.00 & 14.16 minutes. For patient #4, all higher dose volume from 20–30 Gy are larger in GKSRS plan and the lower dose volumes (5–15 Gy) are comparable. CI and T for the HT plans as compared to GKSRS are 0.644 & 0.696 and 36.00 & 36.70 minutes, respectively. For patient #5, all dose volumes except 40 Gy are larger for the HT plans (1.15 to 6.49 times larger). CI and T for the HT plans as compared to GKSRS are 0.547 & 0.507 and 49.00 & 21.00 minutes, respectively. Evaluation of the minimum dose to 100% of the PTV volume shows that this dose is larger in all the GKSRS plans except in patient #5 which are very similar (15.2 Gy vs. 15.4 Gy for GKSRS and HT, respectively). It is possible to improve this dose in the other HT plans by manipulation of the objectives. In turn, manipulation of the objectives in order to increase the minimum dose to 100% of the PTV's volume may result in larger lower iso-dose volumes. Coverage of the PTV for all patients is similar for HT and GKSR (see table 2).

**Table 1:** Conformality Index (CI) of Helical Tomotherapy and Gamma Knife Stereotactic Radiosurgery Plans in Patients with Single Brain Metastasis.

	TOMOTHERAPY	GAMMA KNIFE
Patient #1	0.577	0.597
Patient #2	0.562	0.618
Patient #3	0.603	0.593
Patient #4	0.644	0.696
Patient #5	0.547	0.507

CI = (PTV volume within the prescribed iso-dose line)^2^/[(tumor volume)*(volume of the prescribed dose)]

**Table 2:** Coverage of Helical Tomotherapy and Gamma Knife Stereotactic Radiosurgery Plans in Patients with Single Brain Metastasis.

	TOMOTHERAPY	GAMMA KNIFE
Patient #1	0.983	1.000
Patient #2	0.944	0.990
Patient #3	0.978	0.970
Patient #4	0.962	0.962
Patient #5	0.997	0.997

Coverage = (PTV volume within prescription iso-dose line)/(PTV volume)

**Table 3:** Beam-on Treatment (minutes) Time of Helical Tomotherapy and Gamma Knife Stereotactic Radiosurgery Plans in Patients with Single Brain Metastasis.

	TOMOTHERAPY	GAMMA KNIFE
Patient #1	43.00	50.77
Patient #2	34.00	21.00
Patient #3	30.00	14.16
Patient #4	36.00	36.70
Patient #5	49.00	21.00

**Table 4:** Isodose volumes (cubic centimeter) of Helical Tomotherapy and Gamma Knife Stereotactic Radiosurgery Plans in Patients with Single Brain Metastasis.

Isodose Line	Patient #1		Patient #2		Patient #3		Patient #4		Patient #5	
	Tomo Vol	GK Vol	Tomo Vol	GK Vol	Tomo Vol	GK Vol	Tomo Vol	GK Vol	Tomo Vol	GK Vol

40 Gy	0.009	0.009	0.001	0.001	0.000	0.000	0.000	0.000	0.000	0.000
35 Gy	0.082	0.136	0.039	0.191	0.000	0.000	0.012	0.009	0.136	0.021
30 Gy	0.208	0.332	0.159	0.320	0.028	0.016	0.096	0.138	0.756	0.119
25 Gy	0.430	0.540	0.371	0.431	0.195	0.067	0.277	0.492	1.685	0.689
20 Gy	0.748	0.905	0.709	0.589	0.476	0.225	0.584	0.938	3.093	2.418
15 Gy	1.315	1.417	1.306	0.877	0.961	0.704	1.099	1.665	5.441	4.750
10 Gy	2.851	2.678	2.865	1.545	2.111	1.344	2.363	2.997	11.986	9.339
5 Gy	11.463	7.643	11.394	4.230	8.739	3.774	9.196	8.309	47.574	22.885

Tomo Vol = Tomotherapy Volume.

GK Vol = Gamma Knife Volume.

**Table 5:** Minimum dose (Gy) to 100% of the PTV volume.

	TOMOTHERAPY	GAMMA KNIFE
Patient #1	19.3	22.8
Patient #2	16.4	17.6
Patient #3	12.6	14.1
Patient #4	17	19.1
Patient #5	15.4	15.2

## Discussion

Although the PTV coverage based on CIs are comparable between GKSRS and HT, the volume of low-dose spillage is larger in HT than in GKSRS but comparability of techniques occurs as doses converge at the prescribed dose. Therefore, it is inadequate to perform dosimetric comparison using CI or PTV coverage without evaluating the high and low dose spillage volumes. The clinical importance of the low-dose spillage volumes will be different in individual cases and will need clinical corroboration. The HT single fraction dose-volume toxicity may be prohibitive in treating single or multiple lesions depending on the number and the sizes of the lesions due to the toxicities of overlapping low-dose spillage volumes.

The treatment time for GKSRS depends on the prescribed dose and the strengths of the Cobalt sources. The treatment time for HT ranges from 30–49 minutes in these five patients. The clear trend is that the treatment times are longer in HT even when barring the possible required 1–2 intra-fraction interruptions (HT cannot operate longer than 30 minutes), and in two patients, much longer than GKSRS. This interruption may not apply to all helical tomotherapy units. Minimum dose to 100% of the PTV's volume was also better for GKSRS in four out of the five studied cases. Nevertheless, it is possible to improve on this in the HT plans with a potential increase in the volume of the lower iso-doses.

The inherent property of GKSRS plan is the heterogeneous dose distribution across the PTV. Heterogeneity within the PTV is of benefit in terms of increasing tumor control probability [15]. However, heterogeneity within the PTV is detrimental when its position and extent cannot be "planned" to coincide with tumors and happens to land in normal tissues.

One characteristic of IMRT is the ability to create a dose volume with very high conformality index. For the PTV, similar conformality index can be obtained with GKSRS as well as with HT. So the conformality index comparison is an additional convergence point between the two techniques. Both systems have very good ability to create highly conformal volumetric dose distribution and much will not be gained in this type of study to merely compare conformality index. The second characteristic of IMRT is the ability to create "simultaneous integrated boost"-type of dose distribution. Therefore, creating a structure inside the target as a way of planning to increase heterogeneity in the PTV is not unreasonable as this has been normally done in the clinic. We are demonstrating that GKSRS gives a larger high dose volume to the target than HT. Even when we intentionally create the hot spot in the PTV with HT, we cannot match the kind of high dose volume achievable with GKSRS within the PTV. In the opposite direction as we are moving away from the prescribed isodose surface, we have a smaller low dose volume in GKSRS plan than in HT plan.

Finally, HT uses non-invasive immobilization devices and patients are not sedated. MVCT will need to be taken periodically prior to and during treatment delivery. Whereas the PTV coverage appears comparable to GKSRS, the HT plans assume no patient's movement.

## Conclusion

This study showed the non-dosimetric equivalency between GKSRS and single fraction HT based on dosimetric comparisons, practicality of treatment time and the high level of confidence in PTV coverage for GKSRS over the entire treatment duration due to the use of invasive immobilization device. We demonstrated in our study that the conformality achieved by both GKSRS and HT are quite comparable. However, when we move away from the prescribed isodose surface, we are obtaining a larger high dose volume and a smaller low dose volume with GKSRS in comparison with HT such that the dosimetric and biologic advantages would be expected to be greater with GKSRS rather than with HT. Both HT and GKSRS single fraction dose-volume toxicity may be prohibitive in treating single or multiple lesions depending on the number and the sizes of the lesions. This appears to be less of a problem for GKSRS. Finally, HT can treat a much larger lesion than GKSRS.

## Competing interests

The author(s) declare that they have no competing interests.

## Authors' contributions

YY: Drafted the manuscript and participated in data analysis. JP: Corresponding author, prepared manuscript for submission, created tables and results section, calculated conformality and coverage indices, extracted data from Gamma Knife planning system. CS: Extraction of data from tomotherapy treatment planning systems. ML: Conceived of the study and participated in data analysis. VR: Participated in data analysis and manuscript draft.
